# The β3‐subunit modulates the effect of venom peptides ProTx‐II and OD1 on Na_V_1.7 gating

**DOI:** 10.1002/jcp.31018

**Published:** 2023-04-12

**Authors:** Samantha C. Salvage, Taufiq Rahman, David A. Eagles, Johanna S. Rees, Glenn F. King, Christopher L‐H. Huang, Antony P. Jackson

**Affiliations:** ^1^ Department of Biochemistry University of Cambridge Cambridge UK; ^2^ Department of Pharmacology University of Cambridge Cambridge UK; ^3^ Institute of Molecular Bioscience University of Queensland Brisbane QLD Australia; ^4^ Australian Research Council Centre of Excellence for Innovations in Peptide and Protein Science The University of Queensland Brisbane QLD Australia; ^5^ Department of Physiology, Development and Neuroscience University of Cambridge Cambridge UK; ^6^ Present address: Babraham Research Campus PetMedix Ltd. Cambridge UK

**Keywords:** Na_V_1.7, OD1, pain, ProTx‐II, voltage‐gated sodium channel, β3‐subunit

## Abstract

The voltage‐gated sodium channel Na_V_1.7 is involved in various pain phenotypes and is physiologically regulated by the Na_V_‐β3‐subunit. Venom toxins ProTx‐II and OD1 modulate Na_V_1.7 channel function and may be useful as therapeutic agents and/or research tools. Here, we use patch‐clamp recordings to investigate how the β3‐subunit can influence and modulate the toxin‐mediated effects on Na_V_1.7 function, and we propose a putative binding mode of OD1 on Na_V_1.7 to rationalise its activating effects. The inhibitor ProTx‐II slowed the rate of Na_V_1.7 activation, whilst the activator OD1 reduced the rate of fast inactivation and accelerated recovery from inactivation. The β3‐subunit partially abrogated these effects. OD1 induced a hyperpolarising shift in the *V*_1/2_ of steady‐state activation, which was not observed in the presence of β3. Consequently, OD1‐treated Na_V_1.7 exhibited an enhanced window current compared with OD1‐treated Na_V_1.7‐β3 complex. We identify candidate OD1 residues that are likely to prevent the upward movement of the DIV S4 helix and thus impede fast inactivation. The binding sites for each of the toxins and the predicted location of the β3‐subunit on the Na_V_1.7 channel are distinct. Therefore, we infer that the β3‐subunit influences the interaction of toxins with Na_V_1.7 via indirect allosteric mechanisms. The enhanced window current shown by OD1‐treated Na_V_1.7 compared with OD1‐treated Na_V_1.7‐β3 is discussed in the context of differing cellular expressions of Na_V_1.7 and the β3‐subunit in dorsal root ganglion (DRG) neurons. We propose that β3, as the native binding partner for Na_V_1.7 in DRG neurons, should be included during screening of molecules against Na_V_1.7 in relevant analgesic discovery campaigns.

## 1 INTRODUCTION

**Figure 1:**
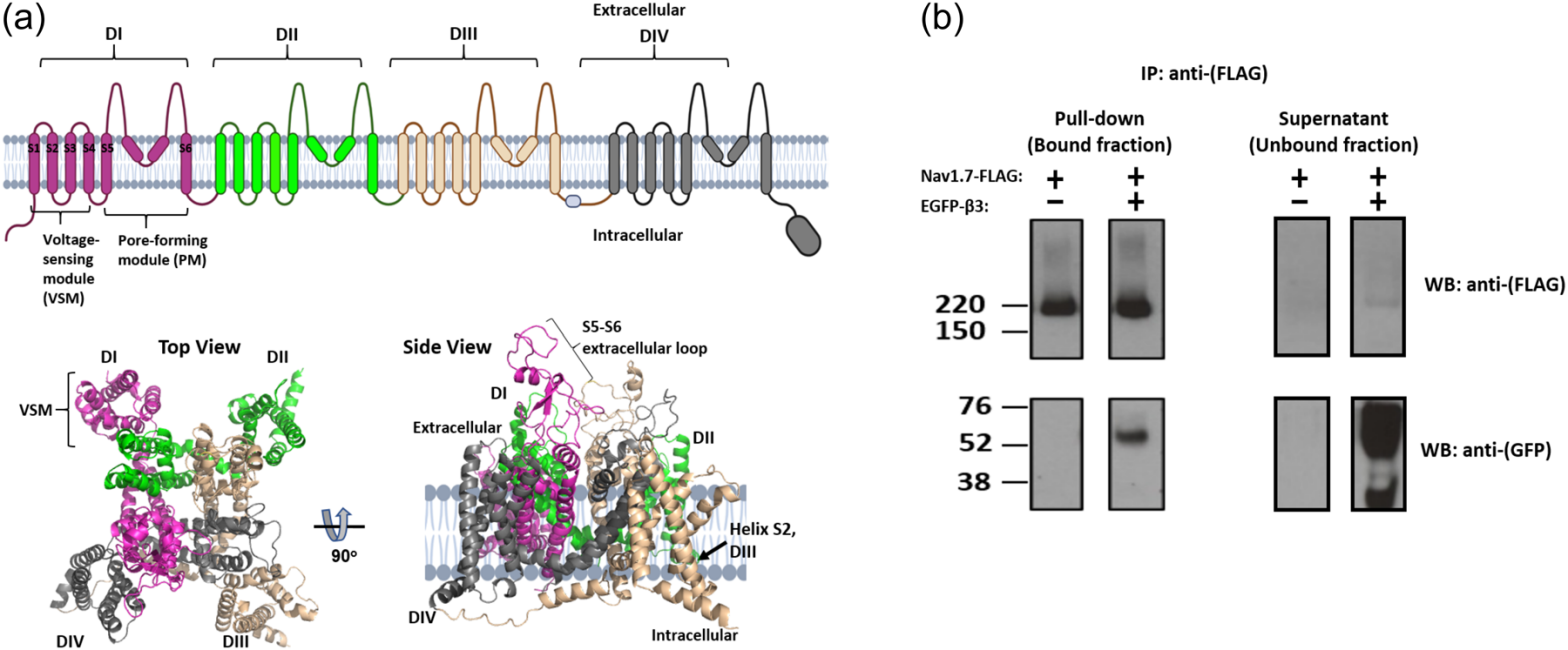
The Na_V_1.7 α‐subunit and its binding to the β3‐subunit. (a) Domain organisation of the human Na_V_1.7 α‐subunit in cartoon form and from the cryo‐EM structure (PDB: 6j8g). The locations of the DI–DIV domains, voltage‐sensor module, pore module, loop regions and transmembrane α‐helices S1–6 are indicated. (b) Stable interaction between FLAG‐tagged Na_V_1.7 α‐subunit and EGFP‐tagged β3‐subunit in HEK293 cells. Cell lysates from Na_V_1.7 or Na_V_1.7‐β3‐subunit cell lines were separately immunoprecipitated with anti‐Flag to pull down Na_V_1.7. Samples were run on SDS‐PAGE gels and blotted for either FLAG (Na_V_1.7) or EGFP (β3‐subunit).

Chronic pain affects up to 20% of individuals worldwide resulting in a large socioeconomic burden (Gaskin & Richard, 2012). Fundamental insights into the mechanisms of pain perception have emerged from patients with congenital pain syndromes. These syndromes are associated with mutations that disable or modify the gating behaviour of some voltage‐gated sodium channel isoforms that notably includes Na_V_1.7, a protein that is expressed on pain‐sensing peripheral neurones (Fischer & Waxman, 2010). The Na_V_1.7 α‐subunit contains four internally homologous domains, DI–DIV, with each domain comprising six transmembrane α‐helices, S1–S6. The S1–S4 helices within each domain form a peripheral voltage‐sensing module (VSM), and the four VSMs surround the central pore‐forming module comprising the S5–S6 helices from each of the four domains (Catterall, 2017) (Figure 1a). However, in dorsal root ganglion (DRG) neurons, the Na_V_1.7 α‐subunit associates with the Na_V_ β3‐subunit (Kanellopoulos et al., 2018). The β3‐subunit consists of a single extracellular immunoglobulin (Ig) domain, a transmembrane alpha‐helical domain and a small, intracellular region. Typically, it induces a depolarising shift in the *V*_1/2_ for steady‐state inactivation of Na_V_ channel α‐subunits (Namadurai et al., 2015; Salvage, Huang, et al., 2020). The Na_V_1.7‐β3 complex, therefore, constitutes an attractive pharmacological target, both in pain management and in experimental pain models.

Animal venoms have proven to be a rich source of various peptidic toxins as Na_V_1.7 modulators that have important therapeutic and experimental applications (Catterall et al., 2007; Robinson et al., 2017). Here, we examine two such peptide toxins: ProTx‐II, a Na_V_1.7 inhibitor from the Peruvian green velvet tarantula *Thrixopelma pruriens* (Middleton et al., 2002) and OD1, a Na_V_1.7 activator from the Yellow Iranian scorpion *Odontobuthus doriae* (Jalali et al., 2005). We identify specific kinetic steps within the Na_V_1.7 gating cycle that are modulated by each toxin and show how these toxin‐modified parameters are themselves affected by the β3‐subunit. We also show that in the absence of the β3‐subunit, OD1 induces a hyperpolarising shift in the *V*_1/2_ of steady‐state activation, leading to an enhanced window current. Our results suggest that the β3‐subunit modulates ProTx‐II and OD1 behaviour in ways that have physiologically important implications and should be taken into account when considering their use in therapeutic or experimental applications.

## 2 MATERIALS AND METHODS

### 2.1 Cell culture and reagents

The previously established stable Na_V_1.7 expressing HEK293 cell line with C‐terminal HAT and FLAG epitope tags (Kanellopoulos et al., 2018), referred to here as HEK293‐Na_V_1.7, was kindly provided by Dr Jing Zhao, University College London. HEK293‐Na_V_1.7 cells were maintained in Dulbecco's modified Eagle's medium (DMEM/F‐12 GlutaMax; Invitrogen; Thermo Fisher Scientific) supplemented with 10% fetal bovine serum (Sigma‐Aldrich) and 400 µg/mL G418 at 37°C and 5% CO_2_. The HEK293‐Na_V_1.7 cells were plated into 100 mm dishes and transfected with 12.5 µg β3‐EGFP (pEGFP‐N1 vector; Yu et al., 2005) and 2.5 µg of a zeocin‐resistant plasmid using polyethyleneimine (PEI; 1 μg/μL) at a PEI:DNA ratio of 3:1. After 48 h, fluorescence was checked by flow cytometry (C6; Accuri) and 200 µg/mL of zeocin was added to the culture medium. Colonies of EGFP‐positive, zeocin and G418‐resistant cells were picked at 72 h post‐transfection and replated. Successful isolates were selected, expanded and stable expression was verified by EGFP expression to generate a stable HEK293 cell line expressing both Na_V_1.7 and the β3‐subunit (Na_V_1.7‐β3).

ProTx‐II was purchased from Alomone Labs and OD1 was synthesised as described previously (Motin et al., 2016). The IC_50_/EC_50_ values were validated for each batch of the toxins.

### 2.2 Co‐immunoprecipitation experiments

The HEK293 cell lines stably expressing Na_V_1.7 and Na_V_1.7‐β3 were separately seeded in 100 mm dishes and grown to ~80%–90% confluency. Cells were washed three times with cold PBS and lysed in 1 mL of lysis buffer (50 mM Tris, 10 mM NaCl, 1% Triton X‐100 (v/v) and 1X protease inhibitor cocktail (Roche, Sigma‐Aldrich)). Lysates were vortexed and mixed by end‐over‐end rotation at 4°C for 30 min and then subject to a clarification step at 10,000*g* for 10 min at 4°C. The clarified lysates were incubated with EZview^TM^ Red FLAG M2 affinity gel (Sigma‐Aldrich) prepared and incubated as per the manufacturer's protocol. The supernatant, nonbound, fractions were transferred to fresh 1.5 mL tubes on ice for downstream analyses. The beads/gel was washed four times in excess cold lysis buffer then subjected to elution in sodium dodecyl sulphate loading buffer and heated to 70°C, 5–10 min. Bound and nonbound fractions were separated on NuPAGE precast gels (Novex, Invitrogen) and transferred to nitrocellulose membrane (iBLOT system; Invitrogen). Membranes were blocked in 5% milk in tris‐buffered saline with 0.1% Tween20 (TBST), then cut into two fractions and incubated with appropriate primary antibody, either mouse anti‐FLAG M2 (Sigma‐Aldrich) or rabbit anti‐GFP polyclonal (GeneTex) and washed four times in 0.1% TBST (10–15 min) and then incubated in corresponding HRP‐coupled secondary antibodies (Bio‐Rad). Amersham^TM^ ECL^TM^ western blot analysis reagents and Hyperfilm were used for chemiluminescent detection of signals. Films were scanned and analysed using ImageJ.

### 2.3 Electrophysiological experiments

Whole‐cell recordings of Na^+^ current (*I*_Na_) were made, as previously described (Salvage, Rees, et al., 2020) either in the absence or presence of 5 nM ProTx‐II or 45 nM OD1, for both Na_V_1.7 and Na_V_1.7‐β3 HEK293 cells. Cells were incubated for a minimum of 10 min before recordings commenced and only recorded under one condition such that the duration of time in the whole‐cell configuration was consistent and reduces any potential time‐dependent effects on seal quality. Control and toxin experiments were performed in a randomised order on different cells, but always within batches on the same day to minimise potential cell–cell variability from different time points in cell culture and passages (e.g., post‐translational modifications etc.). The identity of Na_V_1.7‐β3 expressing cells was always verified by visualisation of EGFP signal on an Olympus IX71 inverted microscope. Experiments were performed in the whole‐cell configuration using an Axopatch 200 amplifier (Axon Instruments) and a Digidata 1322 A digitizer (Axon Instruments), and the Strathclyde Electrophysiology Software Package (WinWCP, Department of Physiology and Pharmacology, University of Strathclyde). The extracellular solution contained (in mM): NaCl 60, KCl 2, CaCl_2_ 1.5, glucose 10, MgCl_2_ 1, CsCl_2_ 90, HEPES 10, pH 7.39 ± 0.02 with NaOH. Patch pipettes with resistance of 1.5–2.5 MΩ were produced from borosilicate glass capillaries (Harvard Apparatus Ltd) using a horizontal puller (P‐87 Sutter Instruments) and filled with intracellular solution, comprising (in mM): NaCl 35, CsF 105, EGTA 10, HEPES 10, pH 7.39 ± 0.02 with CsOH. Signals were sampled at 125 kHz and filtered to 5 kHz using a low‐pass Bessel filter. Only cells with series resistances of 6 MΩ or less, before 75%–80% compensation, were included and leak currents were subtracted using a P/4 protocol. The liquid junction potential (2 mV) was not corrected for. All currents recorded were less than 4.5 nA and data from cells with a current amplitude smaller than 100 pA were removed.

### 2.4 Voltage protocols and kinetic analysis

All voltage protocols used a −120 mV holding voltage of 50 ms duration. The steady‐state inactivation and activation protocol consisted of a 100 ms depolarising pulse ranging from −140 to +45 mV, in 5 mV increments, followed by a fixed −10 mV depolarising pulse of 50 ms duration. Currents elicited from the first pulse constitute activation data and those from the second depolarising pulse provide inactivation data in response to the preceding conditioning pulse. Current traces were normalised against the whole‐cell capacitance (*C*_m_) and the *I/V* relationship plotted from peak current at each test voltage. Values of Na^+^ conductance (*G*_Na_), for families of traces at each test voltage, were determined from the equation

(1) $$G_{\mathrm{Na}}=I_{\mathrm{Na}}/(V-E_{\mathrm{Na}}),$$

where *I*_Na_ is the Na^+^ current and *E*_Na_ is the Na^+^ reversal potential. Peak *G*_Na_ normalised to the maximum *G*_Na_ was plotted as a function of voltage to produce activation curves (*G/G*_max_).

Steady‐state inactivation was determined by normalising *I*_Na_ to the maximum elicited current and plotted against the preceding conditioning voltage to yield inactivation curves. Both activation and inactivation curves were fitted to the Boltzmann function:

(2) $$G/G_{\max}=1/(1+\text{exp}\left(\left(V-V_{½}\right)/k)\right),$$

where *G/G*_max_ is the normalised conductance or current, *V*_½_ is the voltage of half‐maximal activation or inactivation, *k* is the slope factor and *V* is the test voltage or conditioning voltage.

Raw current and voltage data recorded in WinWCP were exported to ASCII text files and analysed for time to peak and *τ* values of single exponential fits of Na channel inactivation/current decay at the point of the fixed −10 mV test pulse following the variable prepulse. It should be noted that for each cell, currents elicited at −10 mV by varying prepulse voltages between −140 and −70 mV, demonstrated consistent current waveforms with respect to times to peak (this can be seen in Supporting Information: Figure 1A lower panels, and was statistically validated via a linear regression fit where the slope, *m*, did not deviate from zero, data not shown). As such, for time to peak, each cell provided up to 15 replicates from which the mean was calculated for each cell. Values of *τ* could similarly be obtained from responses to voltage steps from prepulses ranging from −140 to −100 mV. Here, each cell provided up to nine replicates from which the mean was calculated for each cell. Again, this approach was statistically validated in the same manner as for time to peak. Therefore the *n* number refers to the total number of cells and not the total number of recordings.

Time to peak constitutes the time taken to reach the peak of *I*_Na_ starting from the onset of the depolarising voltage step. This acts as a surrogate for activation rise time which could not be adequately fit to the current rising phase due to the rapid nature of Na_V_1.7 currents which do not always allow for fitting from a fixed baseline due to the capacitance artefact. Thus time to peak provides a more reliable comparison for the effect of the toxins on the activation kinetics of these currents (tight voltage control as maintained by the strict inclusion criteria mentioned above minimises distortion from the voltage‐step, which occurred within 0.02 ms) and has previously been used for measurement of activation kinetics (Lampert et al., 2006). For further qualitative assessment of the rising phase, in the absence of direct measurement, we correlated time to peak with peak current to account for any differences in current magnitude which could influence time to peak and erroneously provide a variation or mask a variation. To fit single exponential functions as follows:

(3) y=−A exp(−t/τ),

the data were first binned by a factor of 5, averaged over 40‐µs intervals (original sampling was every 8 µs or 125 kHz), then smoothed with a moving average to aid in the fitting process. It should be noted that the occasional replicate did not adequately fit the function and so these were excluded.

Recovery from inactivation was examined using a double, P1 and P2, pulse protocol that delivered two identical depolarizing pulses to −10 mV of 50 ms duration. The time interval between P1 and P2 was initially incremented by 1 ms up to 6 ms, followed by 2‐ms increments to 20 ms, then 5‐ms increments to 60 ms, followed by 10‐ms increments to 120 ms and finally 20‐ms increments to 200 ms to ensure enough time was allowed for full recovery and to allow adequate capture of the fast components. Peak currents, *y*, from P2 were normalised to those obtained in response to the conditioning P1 step and plotted against the time intervals. These plots were fitted with a single exponential function as follows:

(4) $$y=A\left(1-\text{exp}\left(-k_{\mathrm{recov}}t\right)\right),$$

where *t* is the time and *k*_recov_ the rate constant of recovery from inactivation. At time 0, the *y* value was set to 0. The curve fit function simultaneously derives a recovery half‐life *t*_½_ from *k*.

### 2.5 Molecular modelling

To predict the binding mode of OD1 to Na_V_1.7, the structure of OD1 (Durek et al., 2013) (PDB id:4HHF) was overlaid onto that of the OD1 homologue AaH2 bound to the deactivated state of the DIV voltage sensing module (VSM4) of human Na_V_1.7 grafted onto the cockroach Na_v_ channel Na_V_PaS backbone (Clairfeuille et al., 2019) (PDB id: 6NT4). The bound AaH2 was then replaced by the overlaid OD1 structure to produce an initial OD1‐human Na_V_1.7 VSM4 complex. To predict the binding mode against the active state of VSM4, OD1 was docked to VSM4 of hNav1.7 grafted onto Na_v_PaS backbone (PDB: 6NT3) (Clairfeuille et al., 2019) using ClusPro (https://cluspro.bu.edu/) and top ranked complex was selected based on the lowest score comprising of electrostatics and van der Waals energy terms (Kozakov et al., 2017). The initial docked complexes were further refined through Rosetta Dock implemented in the ROSIE server (https://rosie.graylab.jhu.edu/) (Lyskov et al., 2013). Finally, the Rosetta‐refined docked complexes were subjected to PRODIGY server (https://wenmr.science.uu.nl/prodigy/) (Xue et al., 2016) to predict the binding affinities.

Electrostatic potential surfaces were calculated using the APBS‐PDB2PQR web server (https://server.poissonboltzmann.org/) (Jurrus et al., 2018) at pH 7, using the PARSE forcefield and visualised in PyMol (Schrödinger, LLC) at the level of ±2 *kT*/*e* using the Adaptive Poisson‐Boltzmann Solver (APBS) plugin 2.1. Figures were generated using PyMol (Schrödinger, LLC).

### 2.6 Data and statistical analysis

Data were analysed using the Strathclyde Electrophysiology Software Package (WinWCP, Department of Physiology and Pharmacology, University of Strathclyde) and custom Python scripts and plotted in GraphPad Prism v.9.1.2. Data are presented as mean ± SEM and statistical analysis performed with two‐way analysis of variance (ANOVA) followed by Sidaks post hoc test on appropriate comparisons (to exclude comparisons of ProTx‐II with OD1). Data and statistical analysis comply with recommendations on experimental design and analysis (Altman, 1982).

## 3 RESULTS

### 3.1 Production of a HEK293 cell line, stably expressing Na_V_1.7‐β3‐subunit complex

The HEK293 cell line stably expressing FLAG‐tagged human Na_V_1.7 has previously been described (Kanellopoulos et al., 2018). In this construct, the FLAG epitope tag does not affect the electrophysiological gating behaviour of Na_V_1.7 (Kanellopoulos et al., 2018). We transfected this cell line with rat EGFP‐tagged β3‐subunit and isolated a HEK293 cell line that stably co‐expressed Na_V_1.7 with β3 (see Section 2). The mature rat and human β3‐subunit sequences differ at only two conservative amino acid positions. Within the extracellular Ig domain—the region responsible for the steady‐state gating shifts (Namadurai et al., 2015; Salvage et al., 2019; Yu et al., 2005)—the sequences are 100% identical (Morgan et al., 2000). Furthermore, as tested in other systems, the rat and human β3‐subunits are functionally interchangeable (Nevin et al., 2021; Salvage et al., 2019). The EGFP tagged β3‐subunit has been extensively used in previous electrophysiological studies of Na_V_ function (Cusdin et al., 2010; Salvage, Rees, et al., 2020; Yu et al., 2005).

Stable co‐assembly of the Na_V_1.7 α‐subunit and β3‐subunit was confirmed by immunoprecipitation (Figure 1b), with a significant amount of β3‐EGFP remaining in the non bound fraction, despite almost all the Na_V_1.7 α‐subunit being precipitated (Figure 1b). This suggests that the β3‐subunit is expressed in excess over the Na_V_1.7 α‐subunit.

### 3.2 Toxin effects on activation and inactivation of Na_V_1.7 and Na_V_1.7‐β3 channels

We tested the effects of ProTx‐II and OD1 on Na_V_1.7 and Na_V_1.7‐β3. ProTx‐II was used at 5 nM and OD1 at 45 nM. These concentrations are comparable with the concentrations used in previous studies (Maertens et al., 2006; Montnach et al., 2021; Schmalhofer et al., 2008). To compare both independent and interacting effects of the toxins with β3 expression, the data were first analysed by two‐way ANOVA, before Sidak's post hoc testing (Tables 1, 2, 3). The *p* values from the two‐way ANOVA for each of the three categories of comparisons; toxin effect, β3 effect and the interaction of these two factors, are reported first, followed by the post hoc analysis, where appropriate. Effects of ProTx‐II were not directly compared with those of OD1.

**Table 1:** Na_V_1.7 ± β3 steady‐state activation and inactivation parameters in the presence and absence of ProTx‐II or OD1.

	Activation				Inactivation		
	Peak *I*_Na_ (pA/pF)	*V*_½_ (mV)	*k*	*n*	*V*_½_ (mV)	*k*	*n*
Na_V_1.7	−33.13 ± 6.792	−17.45 ± 0.925	8.539 ± 0.476	14	−67.01 ± 2.18	−8.108 ± 1.238	14
Na_V_1.7 + ProTx‐II	−18.7 ± 2.06	−20.23 ± 2.608	9.528 ± 0.499	9	−65.35 ± 2.06	−10.31 ± 0.722	9
Na_V_1.7 + OD1	−41.65 ± 10.37	−24.33 ± 2.303*	8.016 ± 0.433	8	−68.53 ± 1.67	−9.387 ± 0.461	8
Na_V_1.7**‐**β3	−74.51 ± 12.94**	−20.3 ± 1.443	7.022 ± 0.385	12	−59.49 ± 0.97*	−9.945 ± 0.198	11
Na_V_1.7**‐**β3 + ProTx‐II	−39.83 ± 7.354^†^	−15.85 ± 1.435	8.06 ± 0.375	7	−55.67 ± 1.64**^#^**	−10.78 ± 0.430	6
Na_V_1.7**‐**β3 + OD1	−85.41 ± 15.53^**^**^	−19.38 ± 1.481	7.132 ± 0.671	7	−63.21 ± 2.96	−9.100 ± 0.422	7

*Note*: Activation and inactivation data are fit to Boltzmann functions with *V*_½_ and *k* derived from these fits. Peak *I*_Na_ is the mean of the absolute maximum *I*_Na_ elicited by each cell during the activation protocol. All data are means ± SEM (*n* ≥ 6, indicated in the table) and compared using two‐way analysis of variance and Sidak's multiple comparison post hoc test.

*p < 0.05 versus Na_V_1.7 and **p < 0.01 versus Na_V_1.7, ^†^ *p* < 0.05 versus Na_V_1.7‐β3, ^#^ *p* < 0.05 versus Na_V_1.7 + ProTx‐II, ^^^ *p* < 0.05 versus Na_V_1.7 + OD1.

**Table 2:** Effect of ProTx‐II and OD1 on Na_V_1.7 ± β3 current kinetic parameters.

	Kinetics		
	Time to peak (ms)	*τ*_fast._ _inact_ (ms)	*n*
Na_V_1.7	0.611 ± 0.034	1.432 ± 0.229	14
+ProTx‐II	0.727 ± 0.054	2.012 ± 0.381	9
+OD1	0.707 ± 0.042	6.013 ± 0.612***	8
Na_V_1.7‐β3	0.637 ± 0.029	1.743 ± 0.369	11
+ProTx‐II	0.836 ± 0.06^**#**^	1.399 ± 0.143	6
+OD1	0.749 ± 0.072	4.164 ± 0.600^**###,†**^	6

*Note*: Time to peak and *τ*_fast.inact_ compared using two‐way analysis of variance with Sidak's post hoc.

***p < 0.001 versus Na_V_1.7, ^#^ *p* < 0.05 and ^###^ *p* < 0.001 versus Na_V_1.7‐β3 and ^†^ *p* < 0.05 versus Na_V_1.7 + OD1.

**Table 3:** Na_V_1.7 ± β3 recovery from inactivation parameters in the presence and absence of ProTx‐II and OD1.

	Recovery from inactivation		
	*k*_recov_	*t*_½_ (ms)	*n*
Na_V_1.7	0.135 ± 0.012	5.580 ± 0.529	11
+OD1	0.165 ± 0.021	4.534 ± 0.681	5
Na_V_1.7‐β3	0.170 ± 0.018	4.376 ± 0.446	7
+OD1	0.260 ± 0.038^**#,†**^	3.008 ± 0.688	6

*Note*: Recovery from inactivation data are fit to a monoexponential function constrained by Y_0_ = 0. *k*_recov_; rate constant of recovery and *t*_½_; recovery half‐life. All data are means ± SEM (*n* ≥ 5, indicated in the table) and compared using one‐way analysis of variance and Sidak's multiple comparison post hoc test.

^#^ *p* < 0.05 versus Na_V_1.7 + OD1, ^†^ *p* < 0.05 versus Na_V_1.7‐β3 + OD1.

**Figure 2:**
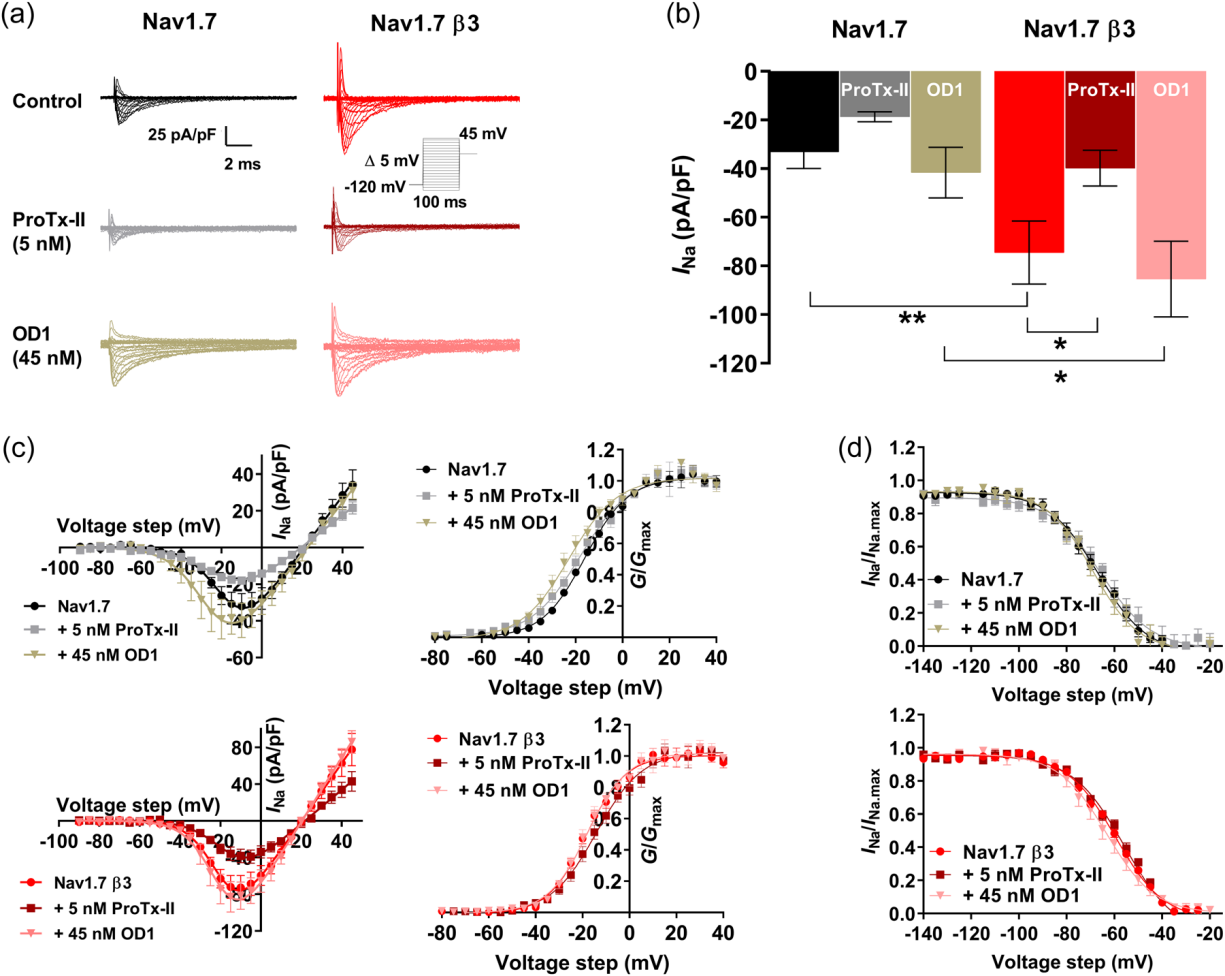
Functional consequences of ProTx‐II and OD1 on Na_V_1.7 steady‐state activation and inactivation with and without the β3‐subunit. (a) Representative whole‐cell Na_V_1.7 and Na_V_1.7‐β3 subunit Na^+^ currents elicited by the activation protocol (inset) in the absence and presence of ProTx‐II or OD1. (b). Histograms of Na_V_1.7 ± β3‐subunit peak current densities (INa) in untreated, ProTx‐II‐treated and OD1‐treated HEK293 cells (compared by two‐way analysis of variance and Sidak post hoc tests). (c) Current‐voltage relationships (left hand panels) and conductance voltage plots (right hand panels) for Na_V_1.7 (top) and Na_V_1.7‐β3‐subunit (bottom), both in the presence and absence of 5 nM ProTx‐II or 45 nM OD1. (d). Channel availability (I_Na_/I_Na.max_) for Na_V_1.7 (top) and Na_V_1.7‐β3‐subunit (bottom), both in the presence and absence of 5 nM ProTx‐II or 45 nM OD1, recorded from a steady‐state inactivation protocol plotted against the conditioning voltage step. All data are mean ± SEM, *n* ≥ 6. The curve fits are to Boltzmann functions (see Section 2) providing the half‐maximal voltages, *V*½ and slope factors, *k* shown in Table 1. *p < 0.05 and **p < 0.01 comparisons as indicated by the bars.

Toxin application and β3 co‐expression exerted independent (*p* = 0.001 and *p* < 0.001, respectively) and noninteracting (*p* = 0.516) effects on peak current (*I*_Na.max_). In the absence of toxin, Na_V_1.7‐β3 expressing cells exhibited significantly larger *I*_Na.max_ than Na_V_1.7 expressing cells (−74.51 ± 12.94 vs. −33.13 ± 6.79 pA/pF, *n* = 12 and 14, respectively, *p* = 0.004), consistent with previous reports (Laedermann et al., 2013; Sokolov et al., 2018). For both Na_V_1.7 and Na_V_1.7‐β3 expressing cells considered collectively, ProTx‐II significantly reduced peak *I*_Na_ (*p* = 0.033). Indeed, ProTx‐II almost halved *I*_Na.max_, although, considered separately, this was only statistically significant for Na_V_1.7‐β3 (*p* = 0.045; Figure 2a,b, and Table 1). This may be due to the data for ProTx‐II treated Na_V_1.7 cells being skewed towards higher values, as some currents fell below the cut‐off threshold. OD1 (45 nM) had no effect on *I*_Na.max_ for both Na_V_1.7 and Na_V_1.7‐β3 expressing cells (Figure 2a,b, and Table 1).

Figure 2c compares *I/V* and *G/V* relationships for Na_V_1.7 and Na_V_1.7‐β3 in the presence and absence of each toxin, derived from the steady‐state activation protocol. Curves were fitted to Boltzmann functions, from which the parameter *V*_½_ and slope factors, *k*, were derived (Table 1). Neither β3co‐expression (*p* = 0.139) nor toxin challenge (*p* = 0.110) independently altered the *V*_½_ of activation. However, there was an interacting effect of the two (*p* = 0.034) (Table 1). OD1 caused a significant hyperpolarising shift in *V*_½_ activation for Na_V_1.7, but only in the absence of the β3‐subunit (Na_V_1.7; −17.45 ± 0.93 vs. Na_V_1.7 + 45 nM OD1; −24.33 ± 2.30 mV, *n* = 14 and 8, respectively, *p* = 0.009). β3 co‐expression (*p* = 0.003) but not toxin challenge (*p* = 0.053), exerted an independent action on the steepness factor of activation *k*. There were no interacting effects (*p* = 0.793) (Table 1). Na_V_1.7‐β3‐subunit steady‐state activation *k* was 7.022 ± 0.385 compared with 8.539 ± 0.476 in Na_V_1.7 channels (*p* = 0.038, *n* = 12 and 14, respectively), resulting in a steeper slope of activation in Na_V_1.7‐β3‐subunit channels. This difference between Na_V_1.7 and Na_V_1.7‐β3‐subunit channels did not persist with either ProTx‐II (*p* = 0.161) or OD1 (*p* = 0.592) (Table 1).

The voltage‐dependence of steady‐state inactivation (Figure 2d) was only affected by β3 co‐expression (*p* < 0.001), and not toxin application (*p* = 0.123) or an interaction of the two factors (*p* = 0.452). The β3‐subunit induced a 7.5 mV depolarising shift of *V*½ inactivation. The slope factor *k* was unaffected by any parameter (Figure 2d and Table 1).

**Figure 3:**
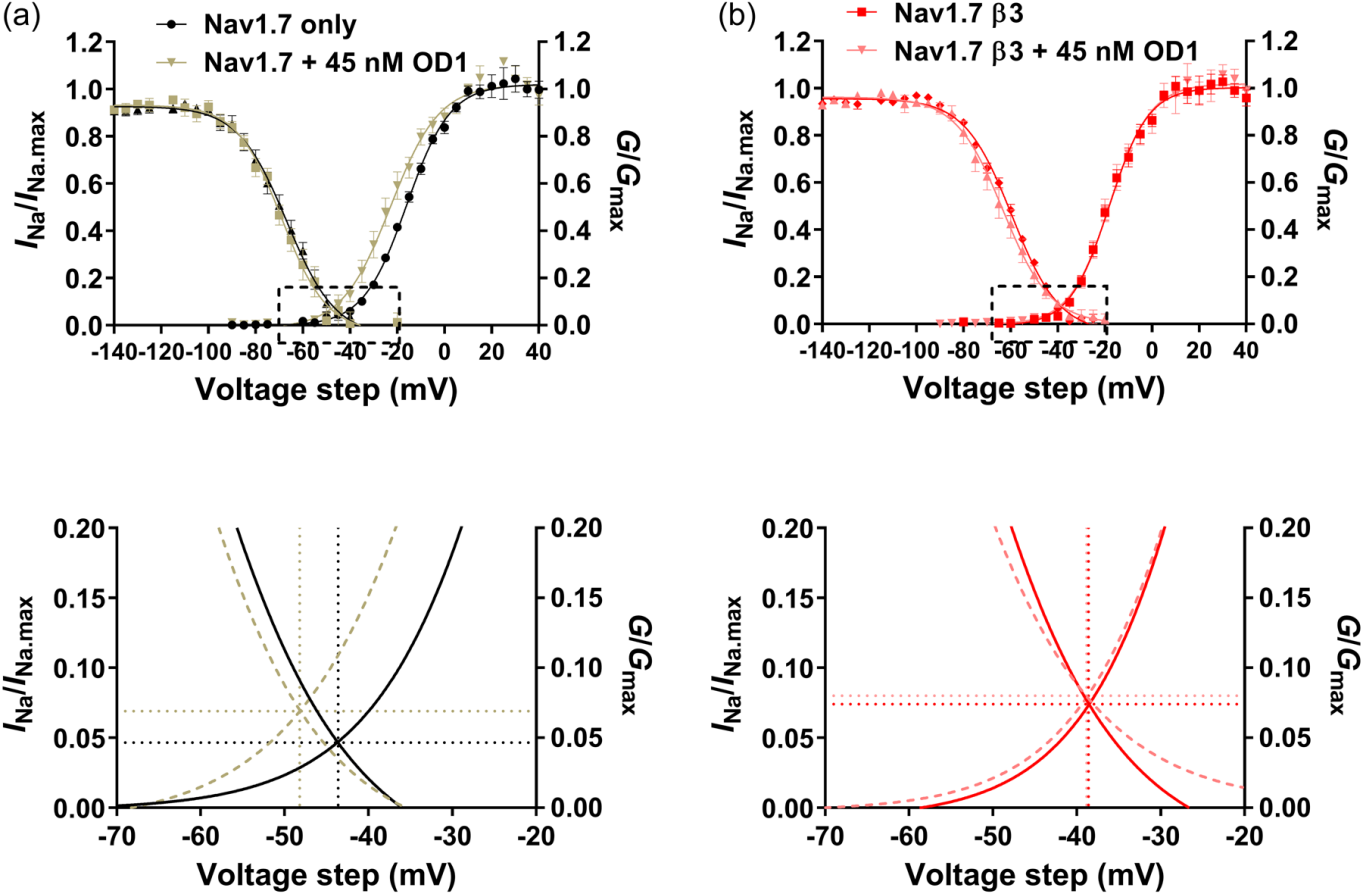
Channel availability and conductance for (a) Na_V_1.7 and (b) Na_V_1.7‐β3‐subunit, with and without OD1. Combined representation of data to demonstrate voltage ranges at which activation and inactivation curves overlap, potentially resulting in window currents. Lower panels show a zoomed in representation of this window, highlighted by a dashed box in the upper panel.

Nevertheless, the distinct actions of OD1 on Na_V_1.7 steady‐state activation, combined with its lack of effect on steady‐state inactivation properties, produced an enhanced and more hyperpolarised window current for OD1‐treated Na_V_1.7 compared with untreated Na_V_1.7. By contrast, OD1 did not alter either the intersection voltage or the channel availability for Na_V_1.7‐β3‐subunit (Figure 3).

**Figure 4:**
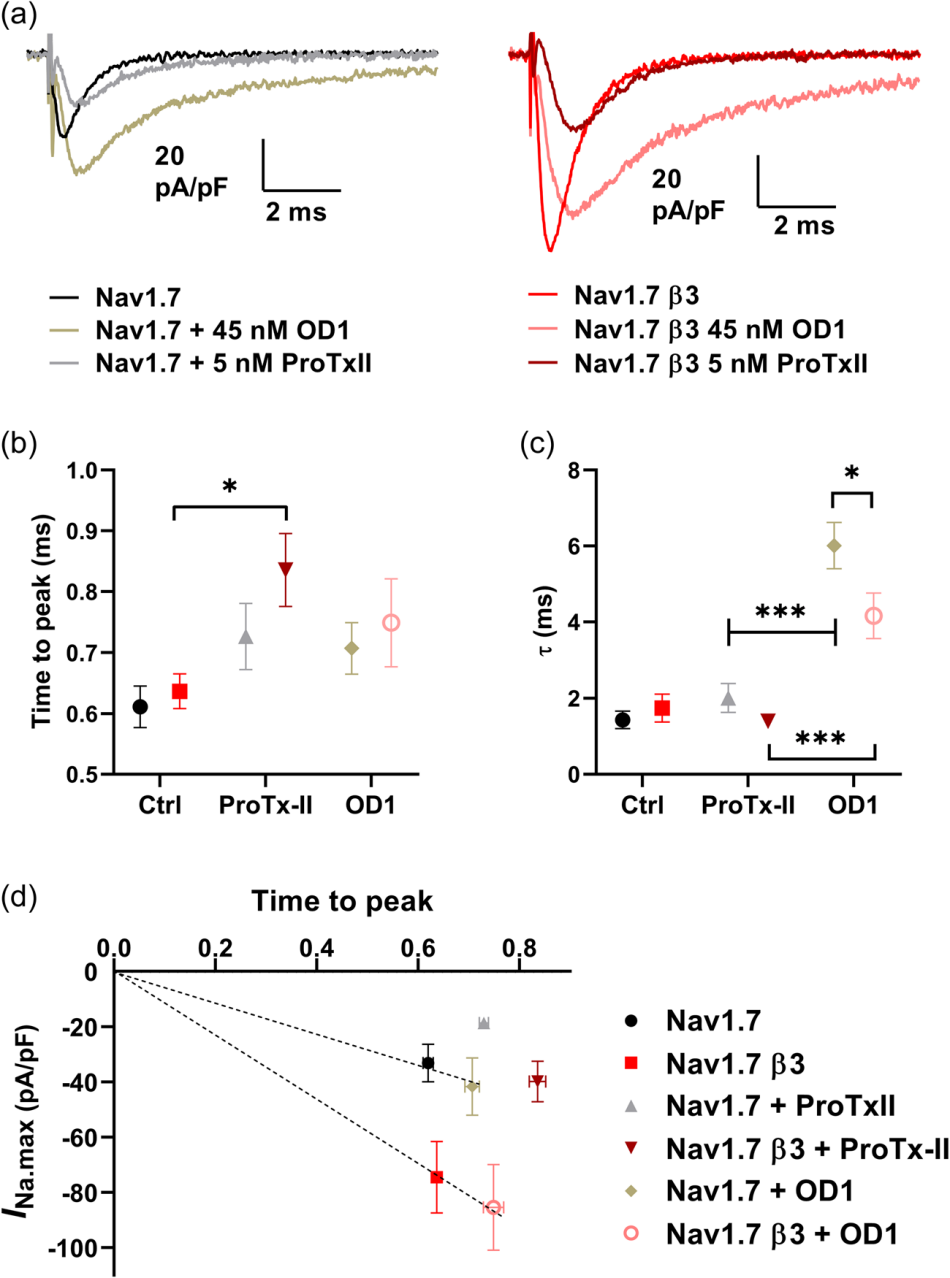
Functional consequences of ProTx‐II and OD1 on Na_V_1.7 and Na_V_1.7‐β3 activation and inactivation kinetics. (a) Representative whole‐cell Na_V_1.7 and Na_V_1.7‐β3 Na^+^ currents in response to the fixed −10 mV test pulse from a variable prepulse, where activation and inactivation kinetics were indistinguishable (−140 to −100 mV) in the presence or absence of ProTx‐II or OD1. (b) Mean time to peak (Na_V_1.7 *n* = 14, Na_V_1.7‐β3 *n* = 11, Na_V_1.7 + ProTx‐II *n* = 9, Na_V_1.7‐β3 + ProTx‐II *n* = 6, Na_V_1.7 + OD1 *n* = 8 and Na_V_1.7‐β3 + OD1 *n* = 6). (c) Mean *τ* values from a single exponential fit to the current decay/inactivation time course (Na_V_1.7 *n* = 14, Na_V_1.7‐β3 *n* = 11, Na_V_1.7 + ProTx‐II *n* = 9, Na_V_1.7‐β3 + ProTx‐II *n* = 6, Na_V_1.7 + OD1 *n* = 8, Na_V_1.7‐β3 + OD1 *n* = 6. (d) Peak currents plotted against time to peak. Data are means ± SEM (b–d) and compared by two‐way ANOVA, followed by Sidak's post hoc test (b and c). *p < 0.05 and ***p < 0.001 comparisons as indicated by the bars.

Given the observable variations of current waveforms (Figure 2a) with different interventions, we investigated kinetic parameters of individual currents elicited from the fixed (−10 mV) test pulse of the activation and inactivation protocol following the variable prepulse (see Section 2). Representative control Na_V_1.7 or Na_V_1.7‐β3 currents for this fixed −10 mV pulse are shown superimposed with their respective toxin interventions in Figure 4a, providing visual clarification of the measured variation in activation and inactivation kinetics. Minimal variation within each condition can be seen by the spread of all data points in both the upper and lower panels of Figure 4b,d, this is anticipated as the voltage‐step eliciting the current is the same (−10 mV). Toxin challenge (*p* = 0.0026) influenced time to peak while β3 co‐expression did not (*p* = 0.132). Consequently, there were no interacting effects (*p* = 0.644) on time to peak (Figure 4b). Na_V_1.7 and Na_V_1.7‐β3 gave indistinguishable times to peak (0.611 ± 0.034 and 0.637 ± 0.029 ms, *n* = 14 and 11, respectively, *p* = 0.951). For both Na_V_1.7 and Na_V_1.7‐β3, this was prolonged by ProTx‐II (0.727 ± 0.054 and 0.836 ± 0.06 ms, *n* = 9 and 6, respectively, *p* = 0.0019 collectively, although individually it was only statistically significant in the presence of β3 (*p* = 0.0111) not in its absence (*p* = 0.0975). OD1 did not significantly increase time to peak in Na_V_1.7 or Na_V_1.7‐β3 cells (0.707 ± 0.042 and 0.749 ± 0.072 ms, *n* = 8 and 6, respectively, *p* = 0.217 and 0.2057) (Table 2). Despite a consistent time to peak between Na_V_1.7 and Na_V_1.7‐β3 channels, the *I*_Na.max_ for Na_V_1.7‐β3 was about two fold greater compared with Na_V_1.7 (Figure 2b). This indicates that Na_V_1.7‐β3 must exhibit a significantly greater rate of sodium current increase compared to Na_V_1.7 alone, as highlighted by plots of times to peak against their corresponding *I*_Na.max_ (Figure 4c). In all conditions, Na_V_1.7‐β3 channels show an increased rate of rise compared with the corresponding Na_V_1.7 condition. Note that gradients did not change in the presence of OD1. Thus, OD1 did not enhance the rate of rise driven by the β3‐subunit. By contrast, ProTx‐II reduced the rate of rise both with and without the β3‐subunit (Figure 4c).

The process of fast inactivation was measured from the time constant (*τ*_fast.inact_), of the exponential current decays (Figure 4d). β3‐subunit co‐expression (*p* < 0.0389) and toxin challenge (*p* < 0.001) exerted both independent and interacting effects (*p* < 0.0325) on *τ*_fast.inact_. Untreated Na_V_1.7 and Na_V_1.7‐β3 gave similar *τ*_fast.inact_ values (1.432 ± 0.229 vs. 1.743 ± 0.369, *n* = 14 and 11, respectively, *p* = 0.888). ProTx‐II did not affect *τ*_fast.inact_ in either Na_V_1.7 (2.012 ± 0.381, *n* = 9, *p* = 0.430) or Na_V_1.7‐β3 cells (1.399 ± 0.143, *n* = 6, *p* = 0.803). In contrast, OD1 markedly increased *τ*_fast.inact_ for both Na_V_1.7 and Na_V_1.7‐β3 (6.013 ± 0.612 and 4.164 ± 0.600, *n* = 8 and 6, respectively, both *p* < 0.001, compared with respective controls), albeit to a significantly greater extent in the absence of β3 (*p* = 0.0172) (Table 2).

### 3.3 Toxin effects on of Na_V_1.7 and Na_V_1.7‐β3 channel recovery from inactivation

**Figure 5:**
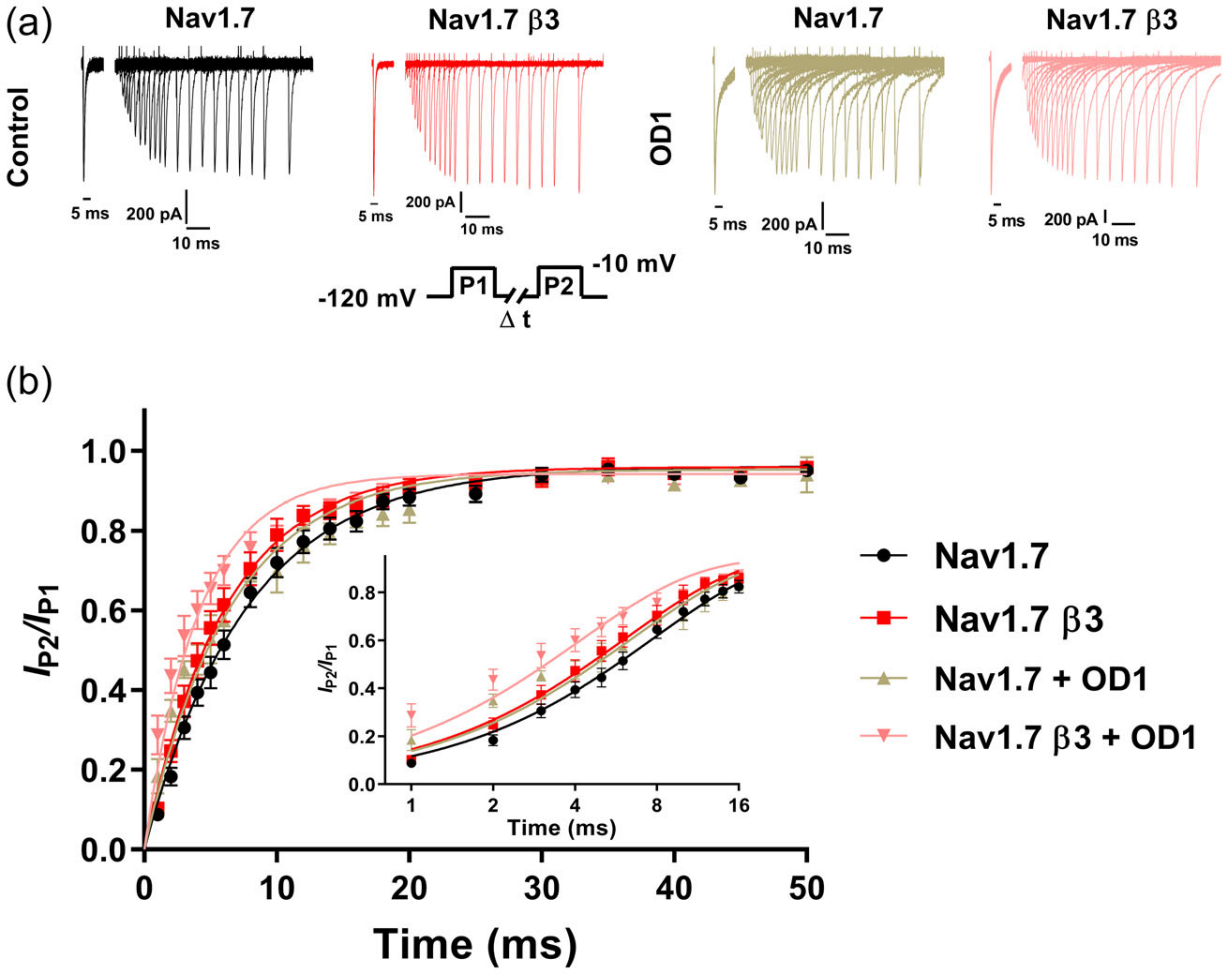
Recovery from inactivation kinetics for Na_V_1.7 and Na_V_1.7‐β3‐subunit, with and without OD1. (a) Typical traces for Na_V_1.7 and Na_V_1.7‐β3 channels in response to a double pulse protocol (inset) to assess recovery from inactivation in the presence and absence of OD1 (only the first 60 ms shown for clarity). (b) Plots of fractional recovery (IP2/IP1) as a function of time. Curves are a single exponential fit to the data providing *k*_recov_ and *t*½. The inset shows the first 16 ms expanded on a logarithmic scale.

Due to the binding site of OD1 at DIV and previous reports that it can accelerate recovery from inactivation (Maertens et al., 2006), the effects of OD1 on recovery were assessed in the absence and presence of β3. A typical double‐pulse protocol (see Section 2 and Figure 5a, inset) was utilised to examine the kinetics of recovery from inactivation for Na_V_1.7 and Na_V_1.7‐β3 with and without OD1. The fraction of current from the second pulse (P2) was normalised to that of the first pulse (P1) and plotted against the varying recovery intervals (Δ*t*) between the two pulses (Figure 5a,b) and fitted to an exponential function. Only the first 50 ms are shown as full recovery is achieved by this time point. The inset further displays the first 16 ms of recovery on a logarithmic time axis highlighting the disparity between recovery kinetics in the presence and absence of OD1 at these early time points (Figure 5b).

Both β3‐subunit co‐expression (*p* = 0.026) and OD1 (*p* = 0.046) influenced recovery half‐times (*t*_½_) but without interaction (*p* = 0.783). However, on post hoc testing, Na_V_1.7‐β3‐subunit and Na_V_1.7 showed similar *t*_½_ before (4.376 ± 0.446 ms, *n* = 7; 5.58 ± 0.529 ms, *n* = 11, *p* = 0.201) and following OD1 challenge (3.008 ± 0.688, *n* = 6, *p* = 0.194, 4.534 ± 0.681 ms, *n* = 5, *p* = 0.1944) (Table 3). Thus, OD1 and the β3‐subunit accelerated recovery from inactivation, but in an independent and nonco‐operative manner. Both β3 co‐expression (*p* = 0.008) and OD1 challenge (*p* = 0.012) independently influenced the rate constants, *k*_recov_ of the exponential fits of the recovery data, with no interaction between the two factors (*p* = 0.190). Na_V_1.7 and Na_V_1.7‐β3 showed similar *k*_recov_ values (0.135 ± 0.012 and 0.170 ± 0.018, *n* = 11 and 7, respectively; *p* = 0.398). However, the application of OD1 significantly enhanced *k*_recov_ for Na_V_1.7‐β3 (0.260 ± 0.038, *n* = 6, *p* = 0.018) but not for Na_V_1.7 channels (0.165 ± 0.021, *n* = 5, *p* = 0.562) (Table 3).

### 3.4 Modelling the putative binding mode of OD1 to human Na_V_1.7

**Figure 6:**
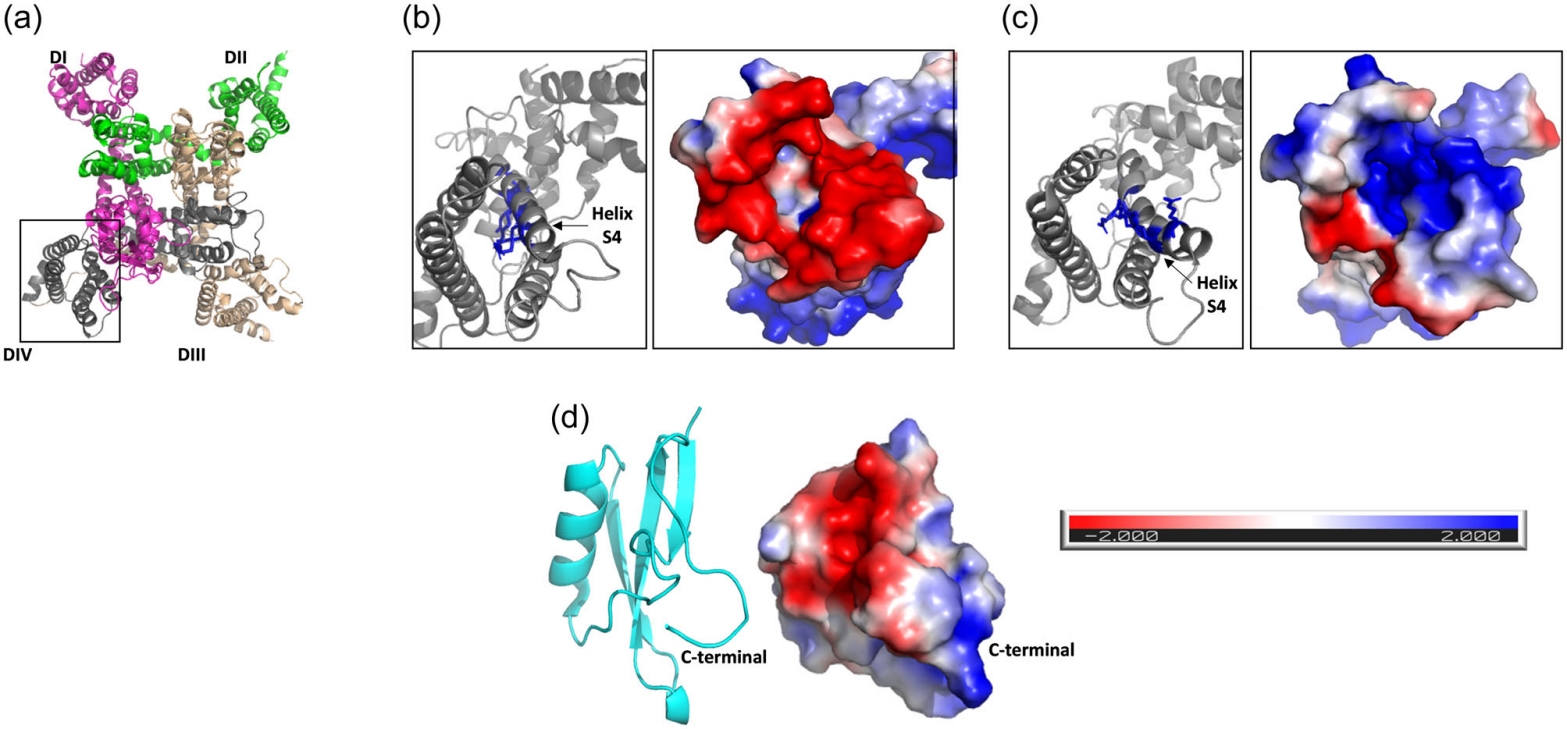
OD1 toxin and its binding site on the Na_V_1.7 DIV voltage sensor module. (a) Top view of human Na_V_1.7 (PDB: 6j8g), as in Figure 1a. The box highlights the DIV voltage‐sensor module (VSM). (b) Enlarged top view of the DIV VSM, in its deactivated state (PDB: 6NT4). (c) Enlarged top view of the DIV VSM, in its activated state (PDB: 6NT3). Left box: cartoon rendering, highlighting the positively charged S4 helix residues. Right box: Electrostatic potential distribution of the solvent‐accessible surfaces. (d) Toxin OD1 (PDB: 4HHF). Left: cartoon rendering. Right: Electrostatic potential distribution of the solvent accessible surfaces. In all cases, electrostatic potentials were calculated by Adaptive Poisson‐Boltzmann Solver in PyMol (https://pymol.org) and visualized in red to blue (−2 to +2 *kT*/*e*).

OD1 is a member of the large family of structurally related scorpion α‐toxins that target the extracellular face of the DIV VSM (Durek et al., 2013; Jalali et al., 2005; Maertens et al., 2006; Motin et al., 2016). In the deactivated state, this site is characterised by a strongly electronegative outer ring. Following membrane depolarisation, the positively charged S4 helix within DIV moves upwards to initiate fast inactivation, so that the outer ring now becomes less electronegative, whilst the inner surface becomes more electropositive (Figure 6a–c) (Clairfeuille et al., 2019). Since the OD1 molecule contains a localised electropositive ʻwedgeʼ in its C‐terminal region (Figure 6d), OD1 could preferentially bind to this site in the deactivated (S4 helix down) state and thus retard the upward movement of the S4 helix.

**Figure 7:**
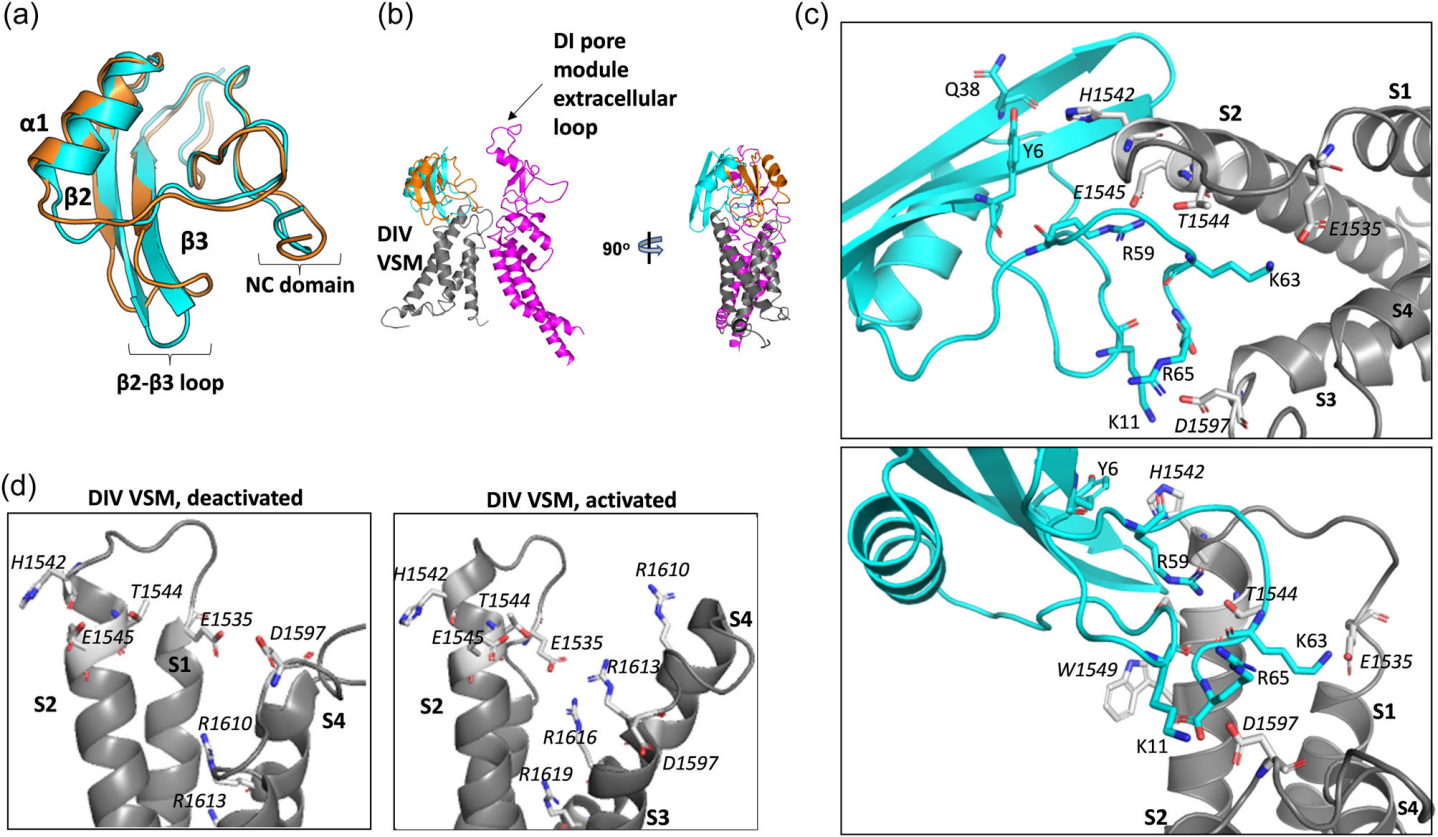
Predicted binding mode of OD1 to DIV voltage‐sensor module (VSM). (a) Close structural similarity between OD1 (cyan) and the related toxin AaHII (gold). (b) Proposed mode of binding of OD1 (cyan) to human DIV VSM (PDB: 6NT4) (grey), overlaid on the previously determined structure of bound AaHII‐toxin (gold) (Clairfeuille et al., 2019). The DI pore module is shown in magenta. (c) Enlarged views of the proposed interface between OD1 and the DIV VSM of human Na_V_1.7 in the deactivated state. Key residues discussed in the text are shown as stick representations and labelled in roman (OD1) or italic (VSM). (d) Enlarged comparison of the DIV, VSMs in the activated state (PDB: 6NT3) and deactivated state (PDB: 6NT4) showing key residues implicated in OD1‐binding (see text for details).

We used in silico protein–protein docking and subsequent pose refinement through energy‐minimisation of the complex (Lyskov et al., 2013) to investigate this question. In our model, OD1 adopts a distinct pose from that of AaH2 (Figure 7b). The interaction with the deactivated state is largely electrostatic. Key features (Figure 7c) are:

(i) A salt‐bridge between E1535 of the VSM S1–S2 loop and K63 of OD1.
(ii) A salt bridge between D1597 of the VSM S3–S4 loop and both K11 and the C‐terminal R65 of OD1.
(iii) A potential salt‐bridge and hydrogen bond between T1544 and E1545 of the VSM and R59 of OD1.
(iv) In addition, we note that H1542 of the VSM lies within ~5 Å from Y6 and Q38 of OD1. It is possible that rotamers of these residues may come into closer proximity and thus provide additional stability.

By contrast, in the DIV, VSM activated state, E1535 now lies close to S4 residues R1613 and R1616 and may even stabilise the upward S4 conformation by salt‐bridge formation to these arginine residues. Similarly, the upward movement of the activated S4 helix induces a large (~9 Å) downward reorientation of D1597, taking it away from any putative OD1‐interaction surface (Figure 7d). Furthermore, as a result of these changes, the extracellular end of the DIV VSM becomes largely electropositive (Figure 6c), which would discourage OD1 (and other relevant scorpion toxins such as AaH2) from forming high affinity interactions, if any binding is at all feasible. In our docking‐based analysis, the predicted pose of OD1 against activated DIV VSM is noticeably drifted from that observed for the deactivated DIV VSM (Supporting Information: Figure 2) with a relevant predicted affinity (Δ*G* = −5 kcal/mol→, *K*_d_ at 25°C 2.2 × 10^−4^M) that appears to be ~100‐fold less than that predicted for the OD1‐deactivated VSM4 complex (Δ*G* = −8.1 kcal/mol→, *K*_d_ at 25°C 1.2 × 10^−6^M). This agrees well with experimental data of AaH2, which is analogous to OD1 (Figure 7a) and manifest ~100 less affinity for the active state of VSM4 (Clairfeuille et al., 2019). Hence, our model can explain the selectivity of OD1 for the deactivated state of the DIV VSM.

## 4 DISCUSSION

Most Na_V_ channels exist in vivo as complexes containing both α and β‐subunits (Namadurai et al., 2015). For the case of Na_V_1.7, proteomic analysis identified the β3‐subunit as a major Na_V_1.7‐binding partner in DRG neurones (Kanellopoulos et al., 2018). Thus, the β3‐subunit should be considered when investigating the pharmacological potential of Na_V_1.7 channel targeted toxins. Here, we compare the effects of the inhibitory spider toxin, ProTx‐II, and the activating scorpion toxin OD1, on Na_V_1.7 gating, in the presence and absence of β3. Our results reveal both independent and interacting effects on kinetic and steady‐state gating parameters.

The Na_V_ β3‐subunit contains a single, extracellular Ig domain, connected to a transmembrane alpha helical domain and a disordered, intracellular C‐terminal region (Namadurai et al., 2015). The location of the β3‐subunit and its orientation on the Na_V_1.7 α‐subunit is not yet known. However, the homologous β1‐subunit binds to the Na_V_1.7 DIII, S2 helix via multiple contacts within its transmembrane domain; and its Ig domain binds to the Na_V_1.7 DI S5–S6 extracellular loop region (Figure 1a) (Shen et al., 2019). Many of these contact residues are conserved between β1 and β3. So it is likely that the β1 and β3‐subunits adopt a similar conformation on Na_V_1.7. This view is supported by the recently determined structure of the β3‐subunit in association with the atypical voltage‐insensitive sodium channel Na_*X*_ (Noland et al., 2022). We observed an increased *I*_Na_ for Na_V_1.7 with the β3‐subunit (Figure 2a,b and Table 1). Taking the peak current and the time to peak together, the β3‐subunit enhanced the rate of activation relative to Na_V_1.7 channel alone (Figure 2b,c). The β3‐subunit did not affect the rate of fast inactivation (Figure 4d and Table 2), or recovery from inactivation (Figure 5a,b and Table 3). The β3‐subunit induced a depolarising shift in the *V*_1/2_ of steady‐state inactivation but did not affect the *V*_1/2_ activation (Figure 2, Supporting Information: Figure 1 and Table 1). These data and the magnitude of the currents obtained with our cell lines are consistent with previous reports of the effects of β3‐subunit on Na_V_1.7 (Laedermann et al., 2013; Montnach et al., 2021; Sokolov et al., 2018) and provide the context with which to interpret the kinetic and steady‐state data obtained with the toxins.

ProTx‐II is a 30‐residue, cysteine‐rich peptide (MW ~3.8 kDa) that binds to an extracellular site on Na_V_1.7 DII, VSM (Bosmans et al., 2008). Hence, the Na_V_1.7 binding sites for ProTx‐II and the Ig domain of the β3‐subunit are likely to be structurally distinct. The bound toxin interferes with the movement of the DII S4 helix, thus inhibiting channel opening (Xu et al., 2019). Our electrophysiological data are broadly consistent with this structural data. For example, ProTx‐II collectively slowed the rate of activation of both Na_V_1.7 and Na_V_1.7‐β3, independent of the β3‐subunit. However, the effect was more marked in the presence of the β3‐subunit (Figure 4b,c and Table 2). The toxin did not affect the rate of fast inactivation, *τ*_fast.inact_ (Figure 4d and Table 2), implying that if a ProTx‐II‐bound channel did activate, then subsequent steps in the channel cycle would be unimpeded. Furthermore, ProTx‐II did not affect the depolarising shift in *V*_1/2_ inactivation induced by the β3‐subunit (Figure 2d and Table 1). Unlike our results, a recent report from Montnach et al. (2021) detected hyperpolarising shifts in both the *V*_1/2_ activation and inactivation, for ProTx‐II on Na_V_1.7. The reasons for this difference are unclear but may reflect the distinct cell‐lines (CHO vs. HEK293) used in the different experiments.

Consistent with previous findings (Maertens et al., 2006), OD1 slowed the rate of fast inactivation (Figure 4). This occurred with both Na_V_1.7 and Na_V_1.7‐β3 (Figure 4d and Table 2). Interestingly, however, the *τ*_fast inactivation_ for OD1‐treated Na_V_1.7 was significantly greater than for Na_V_1.7‐β3 (Table 2). Thus, in the absence of the β3‐subunit, OD1 sustained the channel in the activated state for longer. OD1 also induced a 7 mV hyperpolarising shift in the *V*_1/2_ of activation, but only in the absence of the β3‐subunit (Figure 2c and Table 1). This led to a notable hyperpolarising shift in the window current for the case of Na_V_1.7 relative to Na_V_1.7‐β3 (Figure 3). These OD1‐induced differences in both kinetic and steady‐state parameters may have physiological consequences. The DRG contains distinct classes of pain‐sensing neurones. In particular, the myelinated Aδ fibres transmit fast, sharp and localised pain sensations, whilst the unmyelinated, slower‐conducting C fibres transmit neuropathic pain (Harper & Lawson, 1985). When tested in an ex vivo skin‐saphenous nerve preparation, OD1 preferentially stimulated A fibres (Deuis et al., 2016). This is consistent with its biological function as a defensive mechanism used by the scorpion to deter predators (Jami et al., 2017). Interestingly, although the Na_V_1.7 α‐subunit is expressed throughout the DRG, the β3‐subunit is only expressed in the C‐fibre neurones, where it interacts with Na_V_1.7 at the plasma membrane (Ho et al., 2012; Kanellopoulos et al., 2018; Shah et al., 2000). The resting membrane potential of DRG neurones lies between −48 and −55 mV (Harper & Lawson, 1985), which is within the window current range we observed in our experiments (Figure 3). Thus, the hyperpolarised shift of steady‐state activation and window current, and the increased *τ*_fast inactivation_ exhibited by OD1‐treated Na_V_1.7 (but not Na_V_1.7‐β3), together with the accelerated recovery from inactivation observed at the earlier time points (Figure 5 and Table 3), may make the A fibre Na_V_1.7 channels more susceptible to OD1‐induced depolarisation, relative to Na_V_1.7 channels in C fibres. Since the physiological role of Na_V_1.7 is to amplify transient depolarisations, to the point where other channels such as Na_v_1.8 and Na_v_1.9 can be activated (Dib‐Hajj et al., 2017), the net effect of OD1 would be to preferentially induce repetitive firing in A fibre neurones.

We propose a model to explain how OD1, like AaH2 (Clairfeuille et al., 2019), is likely to manifest a state‐selective interaction in which it preferentially binds to the DIV VSM in the deactivated state (Supporting Information: Figure 2) and thus interferes with the full upward movement of the S4 helix needed for fast inactivation (Figures 6 and 7). In this model, we predict a stabilising salt‐bridge between K11 of OD1 and D1597 on the S3–S4 loop of the VSM (Figure 7c). Interestingly, the OD1 mutation K11V resulted in a three fold loss of potency against Na_V_1.7 and D1597 only adopts a conformation compatible with OD1‐binding in the deactivated state (Figure 7d). An OD1 Y6F mutation significantly reduced potency (Durek et al., 2013). In our model, Y6 could play an additional stabilising role in VSM‐binding (Figure 7c). It has been proposed that channel activation requires the full upward movement of the S4 helices from DI, DII and DIII, together with a partial upward movement of the S4 helix from DIV (Armstrong, 2006). If so, then the trapping of the DIV VSM by OD1 may permit this initial opening, whilst preventing the full movement of the DIV, S4 helix that would normally lead to fast inactivation. This does not in itself explain why OD1 induced a hyperpolarising shift in the *V*_1/2_ of activation. But interestingly, some Na_V_1.7 mutations associated with primary erythromelalgia, including W1538R and S241T, produce a similar hyperpolarising shift in the *V*_1/2_ of activation and also display an enhanced window current (Cregg et al., 2013; Lampert et al., 2006; Yang et al., 2012). However, the shift of *V*_1/2_ activation, seen with W1538R and S241T, in itself may be sufficient to cause hyperexcitability of DRG neurons as it will reduce the threshold for channel activation, and the extent to which this shift occurs may correlate with phenotypic onset. It should be noted that in the cryo‐EM structure of human Na_V_1.7 (PDB: 6j8g) (Shen et al., 2019) (Figure 1a), this tryptophan residue corresponds to W1549 and has been designated as such in Figure 7c. This tryptophan residue is located on helix S2 of the DIV VSM in a region that abuts the OD1‐binding site (Figure 7c). Local perturbation of this region, either by mutation or toxin binding, could influence the steady‐state voltage sensitivity of the initial opening step. The β3‐subunit can modulate the voltage‐sensitivity of DIV S4 movements in Na_V_1.5 via its extracellular Ig domain (Salvage et al., 2019; Yu et al., 2005). As noted above, the β3‐subunit Ig domain binds to the extracellular DI, S5–S6 pore loop (Salvage, Huang, et al., 2020), which in our model lies close to the bound OD1 (Figure 7b). It will therefore be very interesting to determine and examine the structure of the Na_V_1.7‐β3 complex in association with OD1.

In summary, our data emphasise the importance of the β3‐subunit in modulating the responses of Na_V_1.7 to toxic insult and raise the interesting possibility that the pain‐inducing OD1 toxin can selectively modify different subsets of Na_V_1.7 channels, depending on their β‐subunit composition.

## AUTHOR CONTRIBUTIONS

**Samantha C. Salvage**: Conceptualisation; study design; methodology; data acquisition; formal analysis; interpretation; writing—original draft; writing—review and editing. **Christopher L. H. Huang**: Conceptualisation; study design; formal analysis; interpretation; writing—original draft; writing—review and editing. **Antony P. Jackson**: Conceptualisation; study design; formal analysis; interpretation; writing—original draft; writing—review and editing. **Taufiq Rahman**: Study design; methodology; data acquisition; formal analysis; interpretation; writing—original draft; writing—review and editing. **David A. Eagles**: Study design; methodology; data acquisition; formal analysis; interpretation; writing—review and editing. **Glenn F. King**: Study design; formal analysis; interpretation; writing—review and editing. **Johanna S. Rees**: Methodology; data acquisition; writing—review and editing. All authors approved and are responsible for the final version of the manuscript.

## CONFLICT OF INTEREST STATEMENT

The authors declare no conflict of interest.

## Supporting information

Supporting information.
