# Supplementary material for: The β3‐subunit modulates the effect of venom peptides ProTx‐II and OD1 on NaV1.7 gating
Source: J Cell Physiol. 2023 Apr 12;238(6):1354–67. doi: 10.1002/jcp.31018 (PMC10953403; doi:10.1002/jcp.31018)
Supplement: Supplementary file 1 — Supporting information. [file JCP-238-1354-s001.docx]

**Supplementary materials:**


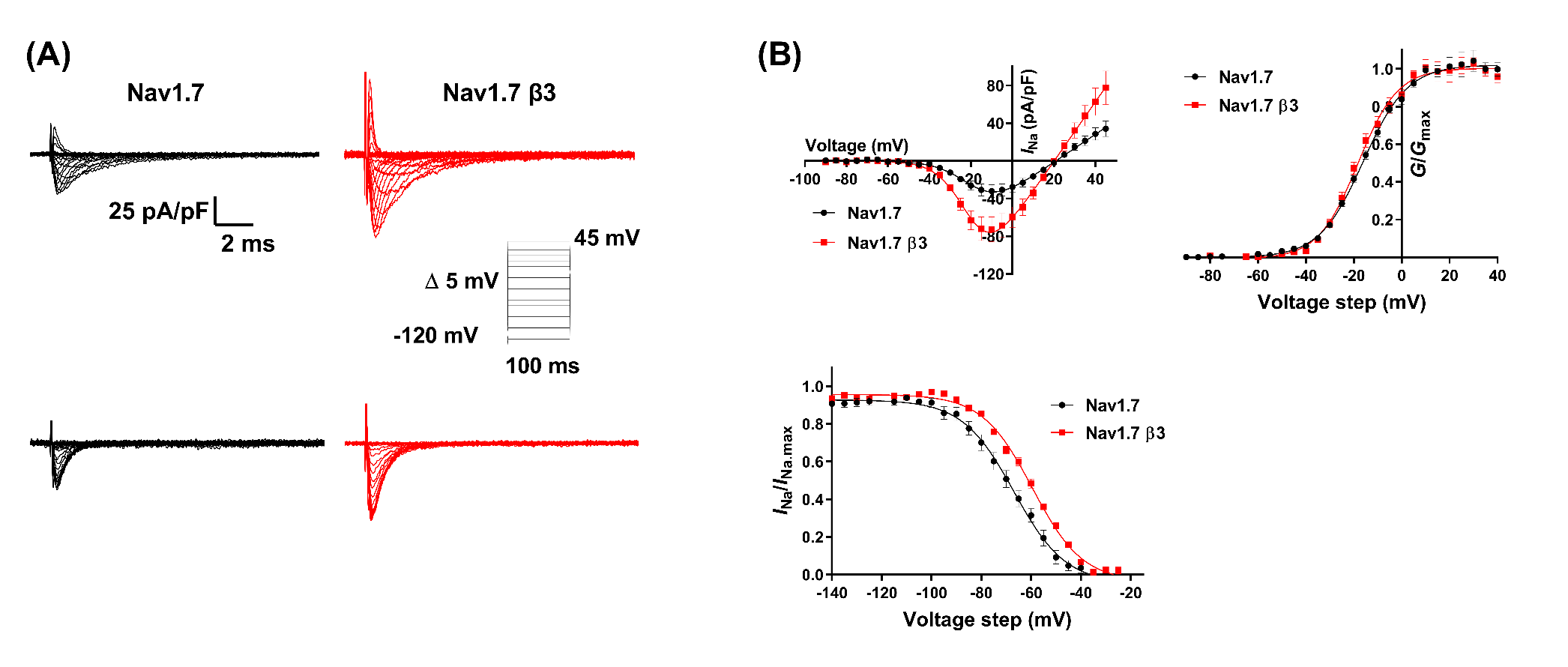


**Supplementary Figure 1. Comparison of steady-state activation and inactivation properties of Nav1.7 and Nav1.7 β3**. A. Representative whole-cell activation and inactivation Na^+^ current traces are shown in the upper and lower panels respectively (Nav1.7 left hand side and Nav1.7 β3 right hand side). B. Accumulated mean ± SEM data for current voltage (top left) and conductance-voltage (top right) relationships as well as channel availability (bottom left).


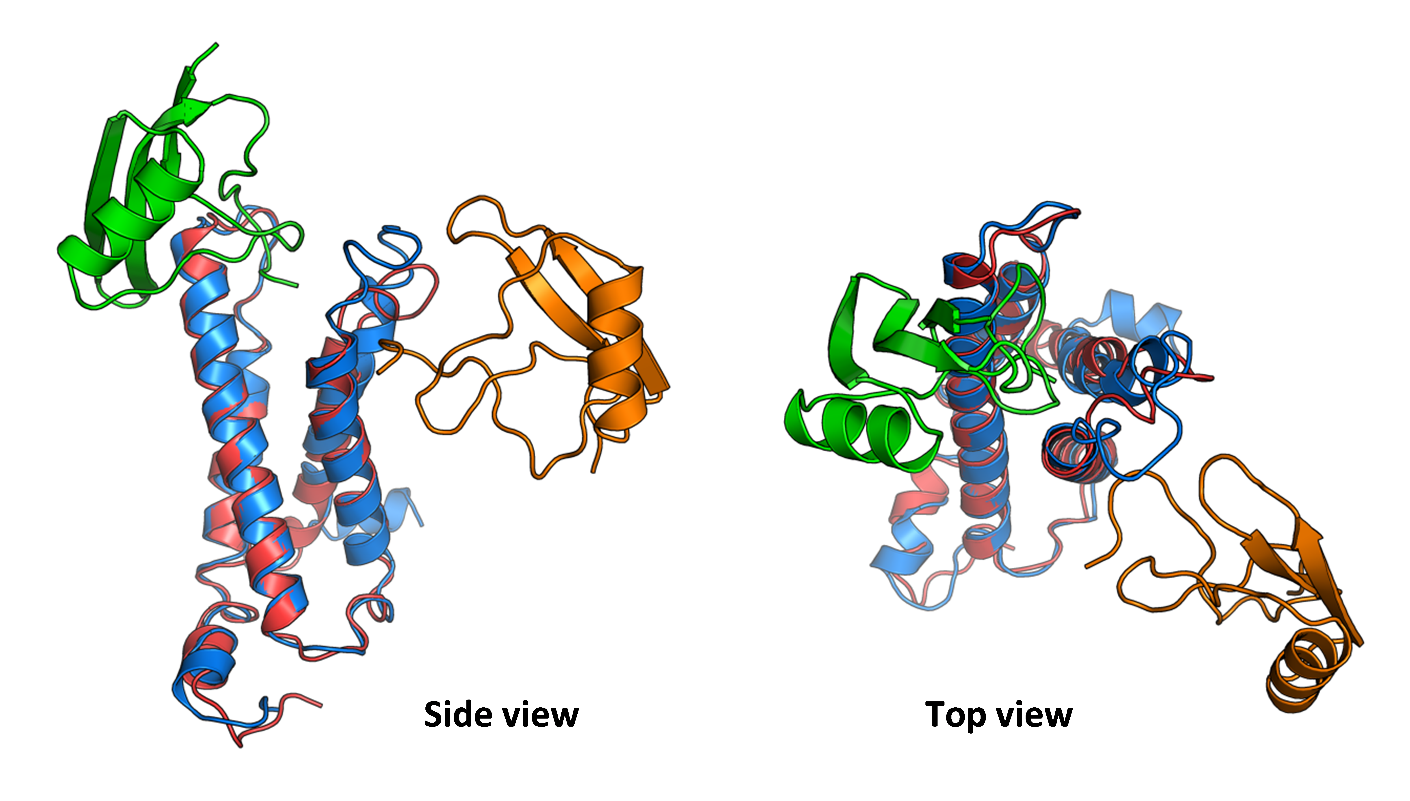


**Supplementary Figure 2. Putative binding poses of OD1 against voltage-sensing module 4 (VSM4) of hNav1.7 at its deactivated (down) and activated (up) positions**. The VSM4 in its down state and the cognate OD1 docked pose are shown as red and green cartoons, respectively. The VSM4 in its up state and the cognate OD1 docked pose are shown as orange and green cartoons, respectively. The VSM4 shown here refers to that of hNav1.7 grafted onto the cockroach Na_v_ channel Na_v_PaS backbone and solved in the activated (PDB: 6NT3, apo form) and deactivated (PDB: 6NT4, AaH2-bound form) states. The poses of OD1 against the two states of the hNav1.7-VSM4 were obtained through protein-protein docking described in section 2.5 (methods). Figures were generated using PyMol (Schrödinger, LLC).
